# Comparison of Extraction Techniques for the Determination of Volatile Organic Compounds in Liverwort Samples

**DOI:** 10.3390/molecules27092911

**Published:** 2022-05-03

**Authors:** Małgorzata Guzowska, Wiesław Wasiak, Rafał Wawrzyniak

**Affiliations:** Faculty of Chemistry, Adam Mickiewicz University in Poznań, Uniwersytetu Poznańskiego 8, 61-614 Poznań, Poland; wasiakw@amu.edu.pl (W.W.); rafwawrz@amu.edu.pl (R.W.)

**Keywords:** Hepaticae, liverworts, specialized metabolites, volatile organic compounds, extraction, *Calypogeia azurea*, hydrodistillation, SLE, MAE, SPME

## Abstract

This article focuses on the comparison of four popular techniques for the extraction of volatile organic compounds (VOCs) from liverworts of the *Calypogeia azurea* species. Since extraction is the most important step in the sample analysis of ingredients present in botanical preparations, their strengths, and weaknesses are discussed. In order to determine the VOCs present in plants, selecting the appropriate one is a key step of the extraction technique. Extraction should ensure the isolation of all components present in the oily bodies of *Calypogeia azurea* without the formation of any artifacts during treatment. The best extraction method should yield the determined compounds in detectable amounts. Hydrodistillation (HD), applying Deryng apparatus and solid-liquid extraction (SLE), microwave-assisted extraction (MAE), and headspace solid-phase microextraction (HS-SPME) were used for volatile extraction. The extracts obtained were analysed by gas chromatography coupled to mass spectrometry (GC-MS) to determine the compounds.

## 1. Introduction

Plants synthesize and secrete many different volatile organic compounds (VOCs). Biologically active substances that are produced by plants are phytochemicals; however, they are not ubiquitous and are the product of specialized metabolism, narrowed down to specific families and species of plants. A feature that distinguishes plant material is the wealth of compounds that are very diverse in terms of physicochemical properties that occur in a very wide range of concentrations. The sample preparation step is important for the reliability of the results of the determined compounds. The extraction of biologically active compounds depends on many factors, including extraction methods, the type of raw material, and extraction solvent. The selectivity of extraction, the number of analytes in the sample, and the ecological aspects are also of key importance [1]. Specialized metabolites may have a wide variety of polarities, solubility, volatility, thermal stability, and the presence of different functional groups. They are often found in low concentrations in the raw material; therefore, there is no universal and simple method to isolate them. Obtaining VOCs is a difficult process, as they make up a small fraction of the plant’s raw material. Various extraction methods are used. The most common method of extracting essential oil from a plant is hydrodistillation (HD). Most often, it is carried out with a glass apparatus of the Clevenger or Deryng type. Both devices are recommended pharmacopoeia devices for determining the content of essential oils. The first is described by the *European Pharmacopoeia* (VI) and the latter according to the *Polish Pharmacopoeia* (VII) [2]. The efficiency of HD is a fairly complex issue. The general disadvantage of distillation methods is that it is difficult to quantitatively determine the essential oil of small amounts of plants, as the yields are typically low [3]. In the publications that have appeared thus far, various methods of extracting VOCs present in liverworts have been used [4,5,6].

The most common extraction methods for liverworts were headspace solid-phase microextraction (HS-SPME) and solid-liquid extraction (SLE) with solvents of different polarities [6]. In this study, SLE was performed with the use of three different solvents: n-hexane, diethyl ether, and methylene chloride. A Likens–Nickerson distillation was also performed for the extraction of VOCs, in which n-pentane was used to collect the extract. Another method of VOC extraction used was HD in a Clevenger apparatus. In this method, n-hexane was used to collect an extract. High temperature, exposure to air, and hot water or hot steam during long distillation times break down many of the unstable aromatic terpenoids.

Due to the small plant samples that can usually be collected, these methods are not used very often. Therefore, comparing the ingredients obtained by the HD process as well as those from solvent extracts is essential to recognize whether they are natural products or compounds formed during the extraction [6].

The percentage of essential oil in the plant substance is often not high, which requires additional work to increase it or select other extraction methods, for example, microwave-assisted extraction (MAE) [7]. HS-SPME [5,7,8,9,10,11,12] and conventional methods, such as SLE, can also be used to extract VOCs [9,10,11]. Modern extraction methods are certainly more efficient and environmentally friendly as very little or no solvents are used [13,14,15]. Polar solvents, such as methanol, ethanol, or ethyl acetate, are used to extract hydrophilic compounds. For the extraction of more lipophilic compounds, dichloromethane or a 1:1 mixture of dichloromethane/methanol is used. In some cases, hexane extraction is used to remove chlorophyll [16,17]. Appropriate extraction measures must be taken to ensure that potentially specialized metabolites are not lost, distorted, or destroyed during the preparation of a plant sample extract [16,18]. The widely used SLE technique is versatile and relatively simple. MAE uses more drastic conditions than the widely used SLE method, which in turn is relatively time-consuming. However, extracts from HD, SLE, and MAE can be stored at low temperatures for a long time and analysed as many times as necessary without quality changes. SPME requires sample preparation indirectly before gas chromatography (GC) analysis. Thus, it does not allow for the extract to be stored. However, it should not be forgotten, that the SPME technique also has many important advantages, such as simplicity, sensitivity, and a remarkably short extraction time. This technique is commonly used to isolate VOCs for qualitative purposes, such as monitoring the changes in volatile substances during storage, volatile substances profile, or metabolomic purposes [19,20,21,22,23].

Liverworts are widespread throughout the world, but most often in the tropics. Liverworts are the ancestors of all land plants and abundantly produce specialized metabolites, including monoterpenes, sesquiterpenes, monoterpenoids, sesquiterpenoids, and aromatic compounds, many of which exhibit notable biological activities, such as the inhibitory effects on allergic contact dermatitis, cytotoxicity, antibacterial, and antifungal activity, anti-insect activity, and antioxidant properties [24,25,26]. On the basis of these statements, it can be concluded that it is very important to develop the best method for the extraction of these compounds from plants. In their natural environment, you can find deciduous and thalli liverworts. A leafy liverwort typically has leaves of two sizes, arranged in three rows along the stem. The larger leaves (called lateral leaves) grow in two rows, along opposite sides of the stem. Most leafy liverworts are prostrate plants and grow along some substrate (e.g., soil, bark, leaves, or rock). The coplanar arrangement of the lateral leaves gives leafy liverworts a “flat” look that is rare in mosses.

Thallose liverworts, which are branching and ribbon-shaped, grow commonly in moist soil or damp rocks, whereas leafy liverworts are found in similar habitats as well as tree trunks in damp woods. The thallus (body) of thallose liverworts resembles a lobed liver, hence, the common name liverwort (‘liver plant’).

Plants are not economically important to humans, but provide food for animals, facilitate the decay of logs, and aid in the disintegration of rocks by their ability to retain moisture.

Most liverworts contain oil bodies, which are intracellular organelles surrounded by a single membrane in which a wide variety of specialized metabolites are synthesized and accumulated, such as terpenes, terpenoids, sesquiterpenes, sesquiterpenoids, and aromatics [15,16,17,18,19,20]. The presence of nitrogen, sulfur, or both nitrogen and sulfur compounds in liverworts is very rare. The most characteristic chemical phenomenon of liverworts is that most sesqui- and diterpenoids are enantiomers of those found in higher plants [6]. Furthermore, the determination of the composition of secondary metabolites that fall under the scope of chemotaxonomy can be one of the methods to help identify taxonomically difficult species [27].

In this study, various techniques were used to extract volatile components from *Calypogeia azurea*. The purpose of the research is to compare the most frequently used methods of extracting volatile organic compounds from liverwort cells. The results allow the provision of information on which extraction technique is most appropriate for this species of liverwort.

The collected information may be useful in further research into other liverworts from the Calypogeia genus.

## 2. Results and Discussion

Twenty-two samples of *Calypogeia azurea* from Poland (Table 1) were analysed for volatile specialized metabolites in the study. Supplementary Tables S1–S7 show the percentage of compounds detected present in liverwort cells. A total of 73 compounds were detected, 42 of which were identified. Depending on the extraction method used, the content of the identified VOCs differed. The study compares four extraction methods: Three (preparative) methods, which used different types of solvents with different polarities (HD, SLE, and MAE), and the non-preparative SPME method. The most common isolation method is SLE, and the extraction efficiency and activity are highly dependent on the type of solvent used. The polarity of the extraction solvent strongly influences the compounds present in the test sample. Therefore, extraction solvents are selected as they are critical to the complex sample matrix. The extraction solvent system is generally selected according to the purpose of extraction, the polarity of the components concerned, the polarity of the undesirable components, the total cost, safety, and environmental concerns [28]. Essential oils, which were prepared with different solvent methods, took different colours. The colour of the oil produced by the HD was slightly blue to purple. The compounds 1,4-dimethylazulene (**53**) (the bold numbers in the brackets refer to the compounds in Tables S1–S7) were responsible for these colours. Colourless solutions were formed during the maceration from n-hexane and turned dark green on extraction with methanol. Methanol is a very good extractant for chlorophylls, especially for resistant vascular plants and algae [29]. In the case of MAE, the extract obtained took the form of a dark brown liquid, regardless of the solvent used.

**Figure 1:**
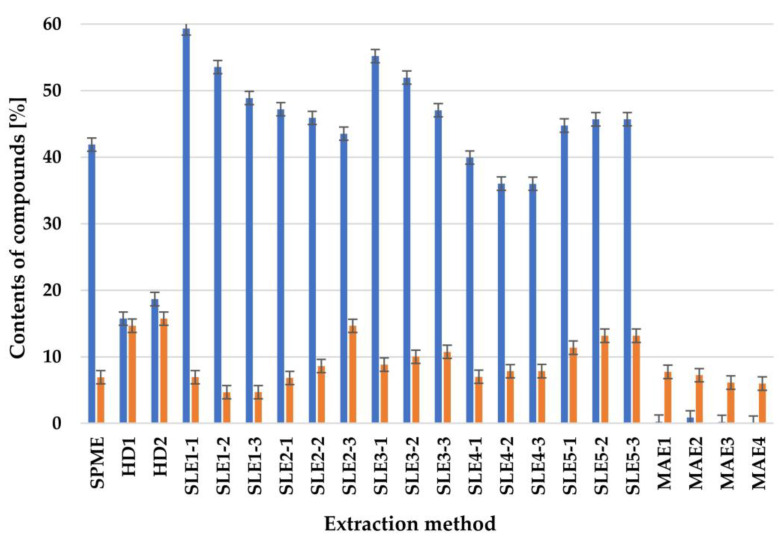
Percentage of 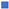
—1,4-dimethyl azulene (**53**) and 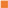
—anastreptene (**18**), depending on the extraction method.

The main component of the *Calypogeia azurea* liverwort is 1,4-dimethylazulene (**53**). This VOC belongs to sesquiterpene hydrocarbons. The relative content of this compound during HD ranged from 16.64% (for n-hexane) to 19.49% (for m-xylene), depending on the solvent used to collect the extract. For this sesquiterpene, the best extraction method is SLE for 24 h n-hexane as the solvent. The relative content of 1,4-dimethylazulene (**53**) for this extraction method is 59.62%. MAE obtained only 1.37–0.21% of this sesquiterpene (Figure 1).

The sum of sesquiterpene hydrocarbons produced is the highest for this research material and ranges from 13.73% to 78.07% of the total essential oil content, depending on the extraction method used. However, for the SPME method, the relative content of 1,4-dimethylazulene (**53**) is 42.67% of all detected VOCs present in liverwort cells. Another sesquiterpene compound, which is also a characteristic chemical compound for this species of liverwort, is anastreptene (**18**). The relative content of this compound ranges from 5.98% for MAE using methanol as a solvent to 15.74% for HD using m-xylene to collect the extract. When this sesquiterpene is detected using the SPME method, the content in liverwort cells is 6.92%.

### 2.1. Comparison of Solvent Extractions

The highest percentage of specialized metabolites could be extracted using the method SLE using n-hexane as the solvent for 24 h (99.51%). MAE, using ethyl acetate as the solvent, was the lowest (55.62%). The solvent method would seem to be the best method used, however, with this method, the amount of solvents used and the extraction time are not favorable. Furthermore, for the HD method and for SLE, the amounts of extracted VOCs were calculated (Table 2). The table shows that the best solvent for the SLE method is methanol, which can yield 0.33 mg/kg of VOCs from 1 g of the sample. In the SLE method, 1 g of liverwort was used and extracted with 10 cm^3^ of solvent. Unfortunately, concentrating the extracted extract to the same volume is a difficult task due to the possible loss of VOCs during the evaporation process. Table 2 shows that the most optimal extraction time for SLE is 48 h, during which the greatest number of specialized metabolites can be isolated, with the exception of n-hexane and methanol. Although, the VOC content does not differ much between the two solvents when extracted within 24 h and 48 h. In the course of the research, the percentage concentrations of isolated VOCs were calculated for the individual extraction methods used in the research. M-xylene and n-hexane were used during HD to collect the extract.

It was found that there were no major differences in the HD between the two solvents. The percentage of sesquiterpenes ranged from 50.95% for n-hexane to 55.25% for m-xylene in the HD, and for aromatic compounds from 22.47% to 24.99%. Because of the lack of significant differences in the percentages of individual groups of VOCs, it seems that it is better to use n-hexane during HD because the oil obtained from plant material can be used for further research, e.g., for the isolation of individual compounds with preparative gas chromatography.

### 2.2. Comparison of HS-SPME with Solvent Methods

HS-SPME is a fairly fast technique to determine VOCs in complex matrices, which are undoubtedly specialized metabolites produced by the oil bodies of *Calypogeia azurea.* Using this technique, it was possible to detect 98.17% of the compounds, including 85.84% identified. As shown in Table 3, a richer composition of VOCs can be obtained using this method (SPME). Compared with the solvent methods, the SPME method can detect a percentage of compounds that are lower, but the number of compounds detected is greater than that of SLE or HD.

For comparison, 66 of the 73 compounds could be detected using HS-SPME. Although only 11 of the 73 compounds were extracted with the SLE method, this may suggest that this method is not a good method for isolating VOCs from *Calypogeia azurea*.

In the case of HD, 53 compounds were detected for n-hexane (HD1) as a solvent, and in the case of m-xylene (HD2), it was 52 out of the 73 compounds that could be isolated with HS-SPME. During the analyzing sample of extracts resulting from the HD and SLE extraction methods, VOCs appeared, which could not be determined by SPME. Examples of such relationships are ledene (**40**), 4,5,9,10-dehydro-isolongifolene (**51**), (+)–spathulenol (**54**). It is sometimes possible to detect compounds that cannot be extracted by solvent methods using HS-SPME. Such compounds include α-pinene (**5**), β-pinene (**6**), and limonene (**7**).

The MAE1-MAE4 appears to be the least effective method of all the others.

Using this method, it is possible to extract 56.71% of the total VOCs for the methanol solvent, and up to 70.09% for the diethyl ether solvent.

Too drastic extraction conditions resulted in the decomposition of some organic compounds, while others, e.g., RI = 1710 (**64**), were extracted in the greatest amount compared to HD, SLE, and MAE. In the case of this method, the conditions were too drastic (exposure to microwave radiation, elevated temperature, and solvent) for a plant with such delicate cell walls.

### 2.3. Statistical Analysis

#### 2.3.1. Comparison of the Extraction Methods with the Solventless Method

Table 3 presents the results of the comparisons using Student’s t-tests for paired samples and Wilcoxon tests, the objective of which was to capture the significance of the differences between the solventless method and individual extraction methods using various solvents.

The results obtained from the difference tests, presented in Table 3, show that there are slight differences between the solvent extraction methods compared to the solvent-free method (SPME).

However, with Cohen’s d indices, which define the magnitude of the observed difference between the mean and the tested sample, it was found that in the SLE method there were effects representing a small (*d* > 0.20) or average difference (*d* > 0.50). In each of the samples in the SLE method, a slightly higher level of volatile compounds was observed compared to that of the method without solvent. On the other hand, no possible differences were observed in the HD and MAE methods (*d* < 0.20). The differential effects obtained, despite the lack of statistical significance, indicated a potentially higher level of volatile compounds in the case of using solvents with the SLE method compared to the control method without the use of a solvent.

In Table 4, Table 5, Table 6 and Table 7, analogues of the Student’s t-tests and Wilcoxon tests were performed with the division into individual volatile compounds. However, because of the occurrence of single measurements in the fields of aliphatic, monoterpene, and monoterpenoid, these were excluded from the analyses. In turn, for sesquiterpenoid (Table 4) and the aromatic compounds (Table 5), only some comparisons were made due to the lack of data in the remaining conditions.

When analyzing the effects obtained in the case of sesquiterpene compounds (Table 4), biased effects were found for the SLE under conditions of SLE5-2 and SLE5-3 compared to SPME (*p* = 0.080), indicating a higher level of sesquiterpene compounds in the extraction of the SLE method under the above conditions. No more biased or statistically significant differences were found; however, possible differences were observed in the sample using Cohen’s d index. The mean level of sesquiterpene compounds did not differ at all in the SPME method compared to HD and SLE under the conditions of SLE4-1, SLE4-2, and SLE4-3 (*d* < 0.20). On the other hand, the other conditions for the SLE possibly showed higher levels of sesquiterpene compounds compared to the solvent-free condition (*d* > 0.20). Furthermore, the fourth method possibly showed a higher severity of sesquiterpene compounds under the MAE2 condition compared to SPME, while under other conditions (MAE1, MAE3, MAE4) the severity of sesquiterpene compounds was slightly lower compared to SPME (*d* > 0.20).

In the case of aromatic compounds (Table 5), no statistically significant differences were found under these comparable conditions. However, it was observed that only in the MAE method, Cohen d values greater than 0.20 were obtained, indicating that there was no possible difference between the means. On the other hand, in the case of HD and SLE, medium-sized difference effects (*d* > 0.50) were found under the tested conditions, which showed a possibly higher level of aromatic compounds compared to the solvent-free method.

As in aromatics, the samples tested for sesquiterpenoid compounds did not show statistically significant differences in the SPME condition (Table 6). However, the differences between the measurements were found to be at least weak in each case (*d* > 0.20). Observing the averages, it was found that a higher level of volatiles is possible in the SLE and MAE for the conditions HD1, HD2, and MAE2 compared to the solventless method. For the MAE conditions, SLE1-2 and SLE1-3 showed lower levels of sesquiterpenoid compounds compared to the SPME condition, while the conditions SLE3-1, SLE3-2, and SLE3-3 showed potentially higher levels of sesquiterpenoid compared to the solvent-free condition.

As in the case of general results, no statistically significant differences were found between the individual extraction methods compared to the solvent-free method in the sample of unidentified compounds (Table 7). However, a biased effect of the difference between SPME and SLE4-1 (*p* = 0.068) was found, indicating a higher level of unidentified compounds in the SPME trial. Furthermore, it was noticed that only in the case of the HD method, Cohen’s d index allowed us to find no difference between the solvent-free method and the HD method (*d* < 0.20). For the SLE and MAE methods, at least a weak difference effect was observed between the SLE1-1 and MAE4 measurements compared to the SPME method (*d* > 0.20). This may mean that unidentified compounds may show a lower intensity level with the third and fourth extraction methods.

#### 2.3.2. Differences between Solvents in Hydrodistillation

**Figure 2:**
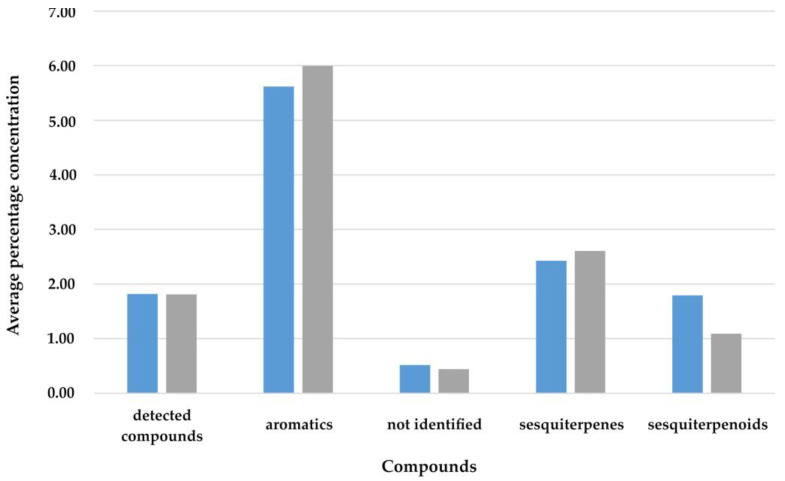
The average levels of volatile compounds using the hydrodistillation 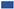
—HD1 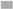
—HD2.

In Figure 2, the mean levels of volatile compounds are presented in the case of using HD. Due to the lack of data, no calculations were performed to compare the compounds of aliphatic, monoterpene, and monoterpenoid using the Student’s t-test for the dependent samples and the Wilcoxon test, which was aimed at confirming the effect obtained.

There were no statistically significant differences between the solvents in HD, *t* (51) = 0.07; *p* = 0.944; *d* = 0.01. Furthermore, the lack of differences was confirmed by the rank test (*p* = 0.722). This means that, regardless of the solvent used, the HD produced the same effect. Based on the analysis with the Wilcoxon test, no significant differences were found in terms of compounds not identified (*p* = 0.398) and sesquiterpene (*p* = 0.163). However, the bias effects of the difference were confirmed for aromatic (*p* = 0.066) and sesquiterpenoid (*p* = 0.068). It turned out that the level of aromatic compounds was slightly higher with the HD2 method, while the level of sesquiterpenoid was higher with the HD1 method in both cases. The effect size index of the difference between the means indicated a difference in the mean value difference (*d* > 0.50).

#### 2.3.3. Differences between Solvents in Extraction Using Solvents

Table 8 presents the results of the analysis of differences in the scope of the SLE method. The analysis had a two-stage character using the Friedman ANOVA test due to the small size of the groups in the measurements.

As it turned out, the analysis of the differences between the groups of solvents in the method with solvents allowed us to find no differences in all the time intervals. This means that regardless of the solvent used, the intensity of the volatile compounds obtained was similar. In the case of differences within the solvent groups, it turned out that there was a border difference effect in the case of the use of methanol (*p* = 0.050), indicating a higher intensity of volatile compounds in the case of the measurement of 24 h than in the case of 48 h and 72 h.

#### 2.3.4. Differences between Solvents in Microwave-Assisted Extraction

**Figure 3:**
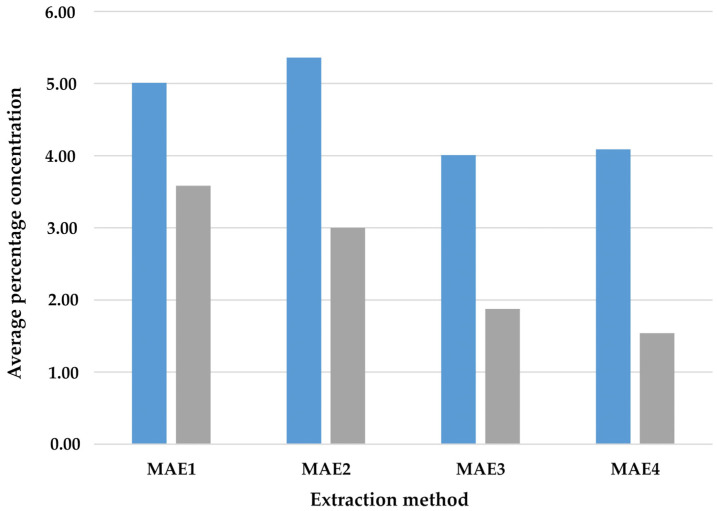
Analysis of variance with the auxiliary Friedman ANOVA versus solvents in Method 4 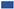
—mean 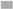
—average rank.

To verify the differences in the fourth method, an analysis of variance was performed in conjunction with an auxiliary Friedman ANOVA test, the results of which are shown in Figure 3.

The analyses performed with the parametric test did not show statistically significant differences in the mean intensity of VOCs, F (3.33) = 1.53; *p* = 0.224; η2 = 0.12. However, the nonparametric analysis, which ignored errors in the measurement of the means, showed a statistically significant effect for the differences between individual solvents in the MAE method, F = 20.01; df = 3; *p* < 0.001. The intensity of VOCs was significantly higher under the MAE1 condition than under the MAE3 (*p* = 0.007) and MAE4 (*p* = 0.001) conditions. Furthermore, a higher level of VOCs was observed under condition MAE2 compared to condition MAE4 (*p* = 0.034). However, no differences were found between the conditions MAE2 and MAE3 (*p* = 0.197), MAE1 and MAE2 (*p* = 1.000) and MAE3 and MAE4 (*p* = 1.000).

## 3. Materials and Methods

### 3.1. Plant Material

The plant material of the *Calypogeia azurea* was collected in 2021 at Szklarska Poręba, latitude, 50°47′52.9″ N; longitude, 15°31′41.8″ E, and the altitude ranged from 700–1200 m ASL. The storage and transfer of plant material were carried out in airtight plastic containers. The collection temperature was 10–12 °C (ambient temperature) and the transfer temperature was 15–16 °C; the pressure was approximately 1013 Mpa (ambient pressure). Only green plants that did not show signs of drying were eligible for collection and further research. In natural habitats, liverwort samples are initially identified on the basis of their morphological structure. The research was carried out on fresh material.

### 3.2. Material and Reagents

The following solvents were used during the research: n-hexane, puriss. p.a., ≥99% (GC), ethyl acetate, puriss. p.a., 99.9% (GC), methanol for GC, Sigma-Aldrich (Steinheim, Germany) and m-xylene, diethyl ether, puriss. ≥99.9% (GC), methylene chloride >99.9%, POCH (Gliwice, Poland). Saturated n-alkanes of C7-C40 standard Supelco (Bellefonte, PA, USA) were used to determine the Kovats retention indices.

Fused silica fibers coated with divinylbenzene/carboxy/polydimethylsiloxane (DVB/CAR/PDMS) (Supelco, Bellefonte, PA, USA) stationary phases were used for the SPME analysis.

Trace 1310 (Thermo Scientific, Waltham, MA, USA) coupled with a mass spectrometer ISQ QD (Thermo Scientific, Waltham, MA, USA) with a 007-5MS column (30 m, 0.25 mm, 0.25 μm) (Quadrex, Woodbridge, CT, USA) were used to analyze the VOC compounds present in the cells of the *Calypogeia azurea* species.

The TriPlus RSH (Thermo Scientific, Waltham, MA, USA) automatic sample injector was used to ensure that the samples were dispensed with sufficient reproducibility.

HD was carried out using a Deryng apparatus consisting of a 500 mL round-bottom flask, a condenser, and a heating bowl (Lab-szkło, Kraków, Poland), recommended by the VI edition of the *Polish Pharmacopoeia* of 2002. The Ethos one (Milestone, Sorisole, Italy) was used for MAE.

### 3.3. Methods

The experimental conditions for the various extraction methods are shown in Table 9.

The specific extraction conditions and methods used in this study are outlined below.

#### 3.3.1. Extraction by Using Headspace Solid-Phase Microextraction

The conditions of sorption and desorption were optimized by selecting the type of stationary phase coated fibers, the amount of biological material, the time, and the temperature. A fresh amount of 5 mg of *Calypogeia azurea* was placed in a screw-capped vial with a 1.7 cm^3^ silicone/Teflon membrane. The vial was then heated at 50 °C and solid-phase microextraction of the headspace was carried out for 60 min. Desorption was performed at 250 °C for 10 min.

#### 3.3.2. Extraction by Using Solvents

An amount of 1 g of plant material was weighed and crushed with an agate mortar and pestle. They were placed in glass bottles and 10 cm^3^ of solvents were added according to the increasing polarity: n-hexane, diethyl ether, methylene chloride, ethyl acetate, and methanol, and were allowed to macerate for 24 h, 48 h, and 72 h. After this time, the solvent was filtered and injected into a GC-MS.

#### 3.3.3. Extraction by Using Microwave-Assisted Extraction

An amount of 5 g of fresh plant material was weighed, placed in Teflon bombs and 50 cm^3^ of diethyl ether was added. The entire process was carried out with the Ethos One microwave-assisted extraction system in 3 steps: ramp time of 10 min to reach 20 °C, a hold time of 20 min at 70 °C, and cooling for 10 min. The final step in the preparation of the sample for analysis was the quantitative transfer of the samples to a 1.7 cm^3^ screw cap vial.

#### 3.3.4. Hydrodistillation Extraction in the Deryng Apparatus

An amount of 5 g of fresh plant material was weighed and placed into a 500 cm^3^ round-bottom flask; we then added 250 cm^3^ of distilled water and 1 cm^3^ of solvent. For HD, two solvents, n-hexane and m-xylene, were used to collect the extract. The sample flask was heated for 3 h after reaching the boiling point. The vapors were condensed by means of a cold refrigerant. After 180 min of extraction to n-hexane, the essential oil was transferred to vials and kept at 5 °C until gas chromatography-mass spectrometry analyses were performed. HD in the Deryng apparatus was carried out according to the *Polish Pharmacopoeia VI* [30].

### 3.4. GC-MS Analysis

The analysis of the composition of the compounds present in the extracts was performed by GC-MS. For liquid samples, the injection volume was 1 µL. The sample was injected in split mode (1:25). Samples analyzed with the SPME technique were injected in splitless mode. The injector temperature in both cases was 250 °C. Helium was used as the carrier gas at a flow rate of 1.0 mL/min. The oven temperature was programmed from 60 to 230 °C at 4 °C/min and then kept isothermal at 230 °C for 40 min. The ISQ QD mass detector was operated at 70 eV in the EI mode in the *m*/*z* range 30–550; transfer line, 250 °C.

The constituents were identified by comparing their MS spectra with those of the literature, reference compounds, computer matching with the NIST 11, and data obtained from the NIST Chemistry WebBook databases, the Mass Finder 4 library, the Adams library databases, and the Pherobase databases [31,32]. The identification of the compounds was verified by Kovats’ retention indices. The Kovats retention indices were determined relative to a homologous series of n-alkanes (C7–C40) under the same operating conditions. The quantitative data of the components were obtained by integrating the TIC chromatogram and calculating the relative percentage of the peak areas. Each sample was analysed three times.

### 3.5. Statistical Analysis

The results obtained from three separate tests were averaged and expressed as a mean ± standard deviation. In order to verify the differences between the extraction methods with respect to the specificity of volatile compounds, statistical analyses were performed using IBM SPSS Statistics 27 software. The statistical methods used included the Student’s t-test for dependent samples and its nonparametric equivalent Wilcoxon test, as well as an analysis of variance for multiple measurements with its nonparametric equivalent Friedman’s ANOVA. Parametric and nonparametric tests were used in parallel as a result of the varying sample sizes of the extraction methods. A threshold of α = 0.05 was used as the significance level.

## 4. Conclusions

The presented studies are the first to concern (VOCs) formed in the oily bodies of *Calypogeia azurea*. These studies demonstrated the advantages and optimal use of commonly used extraction techniques.

Based on the research carried out, it is possible to find that, despite many studies on the effectiveness of individual techniques for the extraction of metabolites from plant material, you cannot find undoubtedly certain and universal information on which of the techniques available today are the most effective in practice. There is no clear information on the scope of the literature on the applications of the described sample/batch preparation procedures for plant material that allows one to maximize the attainable test concentrations of metabolites. Therefore, it is considered advisable to conduct research on the effectiveness of extraction techniques for the most commonly used in order to isolate metabolites from the plant material.

The article compares four methods to extract volatile organic compounds present in the oily bodies of *Calypogeia azurea* liverwort.

On the basis of the conducted experiments, it has been established that one of the best methods of analysis for the determination of VOCs found in the species *Calypogeia azurea* is SPME. Unfortunately, this method can only be used to determine the qualitative composition. SPME extraction allows the identification of low-boiling compounds that co-elute with solvents used in other methods. The main advantage, in addition to simplicity, speed, and low cost, is its “green” character. This technique does not require any toxic solvents, which is especially important nowadays. The SPME method is a method in which the amount of research material is small, which is especially important when analyzing samples, the acquisition of which is quite difficult.

On the contrary, if more research is needed using the essential oils obtained, HD is the best extraction method. With a relatively short extraction time, small amounts of solvents are used. Small amounts of samples used to perform the extraction are beneficial for samples that can be difficult to obtain. During HD, the Deryng apparatus is used, the costs of which are not high, and the extraction costs are low. The disadvantage of this process is undoubtedly the possibility of artifacts forming during heating.

The SLE method gives the greatest relative amount, and one group of compounds, the sesquiterpenes, is a self-absorbing method that uses quite large amounts of solvents. The extracts obtained in this way are diluted too much to give a reliable result when analyzed using GC-MS.

GC-MS analysis allowed for the identification of 43 components, which, depending on the extraction method used, constituted 31.64% to 97.02% of the obtained product. The MAE method was too drastic and resulted in the creation of large amounts of artifacts. Although quick and simple, this extraction technique is too drastic for delicate plants, such as liverworts.

It seems justified to further develop the HD process to obtain essential oils from liverworts. Furthermore, HD is indeed the primary technique and SPME is a complementary method for this type of sample.

## Figures and Tables

**Table 1:** The liverworts sampling data used for studies.

No.	Sample Code	Extraction Method
1	SPME	HS-SPME
2	HD1	Hydrodistillation with n-hexane
3	HD2	Hydrodistillation with m-xylene
4	SLE1-1	Maceration with n-hexane (24 h)
5	SLE1-2	Maceration with n-hexane (48 h)
6	SLE1-3	Maceration with n-hexane (72 h)
7	SLE2-1	Maceration with diethyl ether (24 h)
8	SLE2-2	Maceration with diethyl ether (48 h)
9	SLE2-3	Maceration with diethyl ether (72 h)
10	SLE3-1	Maceration with methylene chloride (24 h)
11	SLE3-2	Maceration with methylene chloride (48 h)
12	SLE3-3	Maceration with methylene chloride (72 h)
13	SLE4-1	Maceration with ethyl acetate (24 h)
14	SLE4-2	Maceration with ethyl acetate (48 h)
15	SLE4-3	Maceration with ethyl acetate (72 h)
16	SLE5-1	Maceration with methanol (24 h)
17	SLE5-2	Maceration with methanol (48 h)
18	SLE5-3	Maceration with methanol (72 h)
19	MAE1	Extraction assisted by microwave radiation with diethyl ether
20	MAE2	Extraction assisted by microwave radiation with methylene chloride
21	MAE3	Extraction assisted by microwave radiation with ethyl acetate
22	MAE4	Extraction assisted by microwave radiation with methanol

**Table 2:** The content of VOCs (mg/kg) depending on the extraction method.

HD	SLE n-Hexane			SLE Diethyl Ether			SLE Methylene Chloride			SLE Ethyl Acetate			SLE Methanol		
	24 h	48 h	72 h	24 h	48 h	72 h	24 h	48 h	72 h	24 h	48 h	72 h	24 h	48 h	72 h
0.15	0.19	0.23	0.24	0.13	0.12	0.09	0.11	0.10	0.09	0.22	0.20	0.20	0.23	0.32	0.33

**Table 3:** Analysis of the differences between the method without solvent and the individual extraction methods among all volatile compounds.

Sample Code	Method with Solvents		Method without Solvent (SPME)		*t*	*p*	*d* Cohen	*p* Wilcoxon
	Mean	SD	Mean	SD				
HD1	1.98	4.88	2.06	6.51	0.11	0.909	0.02	0.852
HD2	1.93	5.09	1.97	6.38	0.06	0.953	0.01	0.987
SLE1-1	9.02	17.90	6.87	12.42	−1.25	0.238	0.38	0.534
SLE1-2	6.10	14.10	4.88	10.60	−1.18	0.258	0.29	0.660
SLE1-3	4.97	12.16	4.14	9.83	−1.08	0.295	0.25	0.446
SLE2-1	13.02	17.72	10.52	14.64	−1.29	0.243	0.49	0.310
SLE2-2	13.53	16.84	10.52	14.64	−1.69	0.143	0.64	0.128
SLE2-3	14.02	15.41	10.52	14.64	−1.68	0.145	0.63	0.128
SLE3-1	12.17	18.48	8.78	14.27	−1.76	0.122	0.62	0.123
SLE3-2	12.22	17.40	8.78	14.27	−1.89	0.100	0.67	0.093
SLE3-3	11.98	15.95	8.78	14.27	−1.93	0.094	0.68	0.123
SLE4-1	8.10	14.57	6.50	11.91	−0.81	0.433	0.23	0.875
SLE4-2	7.05	12.62	5.62	11.18	−0.86	0.406	0.23	0.950
SLE4-3	6.78	11.86	5.62	11.18	−0.83	0.419	0.22	0.875
SLE5-1	10.24	14.63	8.32	13.41	−1.34	0.217	0.45	0.260
SLE5-2	9.65	14.47	7.49	12.91	−1.54	0.158	0.49	0.139
SLE5-3	9.67	14.51	7.49	12.91	−1.52	0.164	0.48	0.139
MAE1	4.02	7.46	3.91	9.99	−0.04	0.972	0.01	0.723
MAE2	3.82	6.34	3.67	10.02	−0.05	0.962	0.01	1.000
MAE3	3.24	6.74	3.78	10.01	0.17	0.864	0.04	0.287
MAE4	3.54	6.64	3.89	10.33	0.11	0.915	0.03	0.605

*t*—Student’s t-test result. *p*—significance level. *d* Cohen—Cohen’s average deviation. *p* Wilcoxon—Wilcoxon’s average deviation.

**Table 4:** Analysis of the differences between the solventless method and individual extraction methods among sesquiterpene compounds.

Sample Code	Method with Solvents		Method without Solvent (SPME)		*t*	*p*	*d* Cohen	*p* Wilcoxon
	Mean	SD	Mean	SD				
HD1	2.62	5.46	2.06	6.51	0.56	0.585	0.13	0.433
HD2	2.86	5.99	1.97	6.38	0.42	0.677	0.10	0.520
SLE1-1	12.43	23.25	6.87	12.42	−1.13	0.310	0.46	0.753
SLE1-2	11.82	20.94	4.88	10.60	−1.17	0.296	0.48	0.917
SLE1-3	9.69	17.96	4.14	9.83	−1.07	0.324	0.41	0.866
SLE2-1	20.46	23.15	10.52	14.64	−1.75	0.223	1.01	0.285
SLE2-2	21.81	20.88	10.52	14.64	−2.91	0.101	1.68	0.109
SLE2-3	25.25	15.89	10.52	14.64	−2.25	0.153	1.30	0.109
SLE3-1	24.74	26.37	8.78	14.27	−2.46	0.133	1.42	0.109
SLE3-2	24.81	23.54	8.78	14.27	−3.12	0.089	1.80	0.109
SLE3-3	23.49	20.42	8.78	14.27	−2.75	0.111	1.59	0.109
SLE4-1	13.71	17.77	6.50	11.91	−0.30	0.782	0.15	0.465
SLE4-2	13.87	15.36	5.62	11.18	−0.18	0.867	0.09	0.465
SLE4-3	14.23	15.24	5.62	11.18	−0.30	0.787	0.15	0.465
SLE5-1	18.12	18.94	8.32	13.41	−1.83	0.164	0.92	0.144
SLE5-2	15.34	18.49	7.49	12.91	−2.00	0.117	0.89	0.080
SLE5-3	15.40	18.54	7.49	12.91	−1.95	0.123	0.87	0.080
MAE1	2.78	3.92	3.91	9.99	0.98	0.371	0.40	0.600
MAE2	3.88	5.04	3.67	10.02	0.77	0.482	0.35	0.500
MAE3	2.70	3.29	3.78	10.01	0.97	0.386	0.43	0.345
MAE4	2.72	3.62	3.89	10.33	0.97	0.386	0.43	0.345

*t*—Student’s t-test result. *p*—significance level. *d* Cohen—Cohen’s average deviation. *p* Wilcoxon—Wilcoxon’s average deviation.

**Table 5:** Analysis of the differences between the solventless method and the individual extraction methods among aromatic compounds.

Sample Code	Method with Solvents		Method without Solvent (SPME)		*t*	*p*	*d* Cohen	*p* Wilcoxon
	Mean	SD	Mean	SD				
HD1	5.62	11.14	3.75	7.43	−1.01	0.387	0.50	0.465
HD2	6.00	11.80	3.75	7.43	−1.03	0.379	0.52	0.285
SLE1-3	11.62	16.38	7.48	10.48	−0.99	0.502	0.70	0.655
SLE4-1	18.89	26.58	7.45	10.52	−1.01	0.498	0.71	0.180
SLE4-2	17.89	25.14	7.45	10.52	−1.01	0.497	0.71	0.180
SLE4-3	15.50	21.67	7.45	10.52	−1.02	0.493	0.72	0.180
MAE3	0.09	0.01	0.10	0.13	0.13	0.921	0.09	0.655

*t*—Student’s t-test result. *p*—significance level. *d* Cohen—Cohen’s average deviation. *p* Wilcoxon—Wilcoxon’s average deviation.

**Table 6:** Analysis of the differences between the solventless method and the individual extraction methods among sesquiterpenoid compounds.

Sample Code	Method with Solvents		Method without Solvent (SPME)		*t*	*p*	*d* Cohen	*p* Wilcoxon
	Mean	SD	Mean	SD				
HD1	2.19	3.61	0.26	0.11	−1.05	0.373	0.52	0.273
HD2	1.39	2.03	0.26	0.11	−1.07	0.364	0.53	0.273
SLE1-2	0.20	0.17	0.26	0.13	0.52	0.655	0.30	1.000
SLE1-3	0.18	0.14	0.26	0.13	0.68	0.565	0.39	0.593
SLE3-1	1.48	1.29	0.23	0.16	−1.21	0.439	0.86	0.180
SLE3-2	1.40	1.48	0.23	0.16	−1.01	0.497	0.71	0.180
SLE3-3	1.41	1.55	0.23	0.16	−0.98	0.508	0.69	0.655
MAE2	9.54	13.04	0.23	0.16	−1.00	0.501	0.71	0.655

*t*—Student’s t-test result. *p*—significance level. *d* Cohen—Cohen’s average deviation. *p* Wilcoxon—Wilcoxon’s average deviation.

**Table 7:** Analysis of the differences between the solvent-free method and individual extraction methods among unidentified compounds.

Sample Code	Method with Solvents		Method without Solvent (SPME)		*t*	*p*	*d* Cohen	*p* Wilcoxon
	Mean	SD	Mean	SD				
HD1	0.55	1.15	3.49	9.49	0.19	0.855	0.04	0.983
HD2	0.46	0.77	3.49	9.49	0.53	0.603	0.12	0.872
SLE1-1	1.25	0.53	9.16	16.25	0.35	0.787	0.25	0.655
SLE1-2	0.55	0.57	9.16	16.25	0.92	0.409	0.41	0.225
SLE1-3	0.48	0.44	7.92	15.19	1.02	0.353	0.42	0.345
SLE2-1	0.76	0.08	17.73	20.96	1.77	0.327	1.25	0.180
SLE2-2	0.87	0.39	17.73	20.96	2.09	0.284	1.48	0.180
SLE2-3	0.73	0.59	17.73	20.96	2.67	0.228	1.89	0.180
SLE3-1	0.85	0.04	16.49	22.22	1.65	0.347	1.17	0.180
SLE3-2	0.90	0.07	16.49	22.22	1.64	0.349	1.16	0.180
SLE3-3	0.91	0.08	16.49	22.22	1.64	0.349	1.16	0.180
SLE4-1	0.74	0.52	13.40	19.18	2.26	0.109	1.13	0.068
SLE4-2	0.64	0.43	13.40	19.18	1.68	0.153	0.69	0.116
SLE4-3	0.57	0.41	13.40	19.18	2.01	0.101	0.82	0.116
SLE5-1	0.59	0.36	13.46	19.13	1.45	0.283	0.84	0.109
SLE5-2	0.74	0.39	10.77	17.62	1.40	0.297	0.81	0.109
SLE5-3	0.74	0.39	10.77	17.62	1.40	0.297	0.81	0.109
MAE1	4.47	9.14	9.67	15.96	−0.96	0.373	0.36	0.398
MAE2	3.74	6.63	10.73	17.61	−1.04	0.339	0.39	0.612
MAE3	3.29	8.34	10.85	17.56	−0.63	0.555	0.24	0.398
MAE4	3.13	7.35	10.91	17.52	−0.80	0.448	0.28	0.889

*t*—Student’s t-test result. *p*—significance level. *d* Cohen—Cohen’s average deviation. *p* Wilcoxon—Wilcoxon’s average deviation.

**Table 8:** Analysis of differences in the scope of the SLE method.

Time	24 h			48 h			72 h			Intragroup Tests
Solvents	*M*	*Me*	*SD*	*M*	*Me*	*SD*	*M*	*Me*	*SD*	
n-hexane	8.29	0.66	17.25	5.77	0.35	13.72	4.74	0.29	11.88	*F* = 0.19; *df* = 2; *p* = 0.909
diethyl ether	11.44	3.92	17.01	11.87	5.05	16.29	12.29	8.07	15.08	*F* = 2.67; *df* = 2; *p* = 0.875
methylene chloride	12.17	5.61	18.48	12.22	6.23	17.40	11.98	6.62	15.95	*F* = 3.68; *df* = 2; *p* = 0.159
ethyl acetate	7.52	1.12	14.11	6.62	0.73	12.27	6.38	0.85	11.53	*F* = 3.22; *df* = 2; *p* = 0.199
methanol	9.24	1.38	14.15	8.78	1.11	14.02	8.80	1.11	14.06	*F* = 6.00; *df* = 2; *p* = 0.050
tests between groups	*F* = 2.20; *df* = 4; *p* = 0.699			*F* = 3.35; *df* = 4; *p* = 0.500			*F* = 2.72; *df* = 4; *p* = 0.606			

*M*—mean. *Me*—median. *F*—the result of the analysis of variance. *df*—degrees of freedom. *p*—significance level.

**Table 9:** Experimental conditions used during the extraction of the *Calypogia azurea*.

	SPME	HD	SLE	MAE
the amount of plant material [g]	0.005	5	5	5
solvents	not applicable	water/n-hexane water/m-xylene	n-hexane diethyl ether ethyl acetate, dichloromethane ethanol methanol	diethyl ether
temperature	50/250 °C	100 °C	room temperatures	20–70 °C
time	60 min of sorption 10 min desorption	3 h	24 h 48 h 72 h	40 min
volume of solvent required	not applicable	250 cm^3^ H_2_O/0.5 cm^3^ organic solvent	50 cm^3^	50 cm^3^

## Data Availability

Not applicable.

## Supplementary Materials

The following supporting information can be downloaded at: https://www.mdpi.com/article/10.3390/molecules27092911/s1, Table S1: Volatile compounds detected in the samples analysed by SPME and HD; Table S2: Volatile compounds detected in the samples extracted by n-hexane; Table S3: Volatile compounds detected in the samples extracted by diethyl ether; Table S4: Volatile compounds detected in the samples extracted by methylene chloride; Table S5: Volatile compounds detected in the samples extracted by ethyl acetate; Table S6: Volatile compounds detected in the samples extracted by methanol; Table S7: Volatile compounds detected in the samples analysed by MAE.

## References

[1] Tiwari B.K. (2015). Ultrasound: A Clean, Green Extraction Technology. TrAC-Trends Anal. Chem..

[2] Baj T., Sieniawska E., Kowalski R., Wesołowski M., Ulewicz-Magulska B. (2015). Effectiveness of the Deryng and Clevenger-Type Apparatus. Acta Pol. Pharm.-Drug Res..

[3] Ormeño E., Goldstein A., Niinemets Ü. (2011). Extracting and Trapping Biogenic Volatile Organic Compounds Stored in Plant Species. TrAC-Trends Anal. Chem..

[4] Asakawa Y. (2017). The Isolation, Structure Elucidation, and Bio-and Total Synthesis of Bis-Bibenzyls, from Liverworts and Their Biological Activity. Nat. Prod. Commun..

[5] Komala I., Ito T., Yagi Y., Nagashima F., Asakawa Y. (2010). Volatile Components of Selected Liverworts, and Cytotoxic, Radical Scavenging and Antimicrobial Activities of Their Crude Extracts. Nat. Prod. Commun..

[6] Asakawa Y., Ludwiczuk A. (2013). Bryophytes: Liverworts, Mosses, and Hornworts: Extraction and Isolation Procedures. Methods Mol. Biol..

[7] Chemat S., Lagha A., AitAmar H., Bartels P., Chemat F. (2004). Comparison of Conventional and Ultrasound-Assissted Extraction of Carvone and Limonene from Caraway Seeds. Flavour Fragr. J..

[8] Hallingbäck T., Hodgetts N.G., IUCN/SSC Bryophyte Specialist Group, IUCN-The World Conservation Union, Sveriges lantbruksuniversitet, ArtDatabanken (2000). Mosses, Liverworts, and Hornworts: Status Survey and Conservation Action Plan for Bryophytes.

[9] Garrido A., Ledezma J.G., Durant-Archibold A.A., Allen N.S., Villarreal A.J.C., Gupta M.P. (2019). Chemical Profiling of Volatile Components of the Gametophyte and Sporophyte Stages of the Hornwort *Leiosporoceros Dussii* (Leiosporocerotaceae) From Panama by HS-SPME-GC-MS. Nat. Prod. Commun..

[10] Sakurai K., Tomiyama K., Kawakami Y., Yaguchi Y., Asakawa Y. (2018). Characteristic Scent from the Tahitian Liverwort, *Cyathodium foetidissimum*. J. Oleo Sci..

[11] Ghani N.A., Ludwiczuk A., Ismail N.H., Asakawa Y. (2016). Volatile Components of the Stressed Liverwort *Conocephalum Conicum*. Nat. Prod. Commun..

[12] Rowan D.D. (2011). Volatile Metabolites. Metabolites.

[13] Jha A.K., Sit N. (2022). Extraction of Bioactive Compounds from Plant Materials Using Combination of Various Novel Methods: A Review. Trends Food Sci. Technol..

[14] Madani A., Mazouni N., Nedjhioui M. (2021). Plants’ Bioactive Metabolites and Extraction Methods.

[15] Huie C.W. (2002). A Review of Modern Sample-Preparation Techniques for the Extraction and Analysis of Medicinal Plants. Anal. Bioanal. Chem..

[16] Sasidharan S., Chen Y., Saravanan D., Sundram K.M., Yoga Latha L. (2011). Extraction, Isolation and Characterization of Bioactive Compounds from Plants’ Extracts. Afr. J. Tradit. Complement. Altern. Med..

[17] Cos P., Vlietinck A.J., Berghe D.V., Maes L. (2006). Anti-Infective Potential of Natural Products: How to Develop a Stronger in Vitro “Proof-of-Concept”. J. Ethnopharmacol..

[18] Fabricant D.S., Farnsworth N.F. (2001). The Value of Plants Used in Traditional Medicine for Drug Discovery. Environ. Health Perspect..

[19] Majcher M., Jeleń H.H. (2009). Comparison of Suitability of SPME, SAFE and SDE Methods for Isolation of Flavor Compounds from Extruded Potato Snacks. J. Food Compos. Anal..

[20] Routray W., Orsat V. (2012). Microwave-Assisted Extraction of Flavonoids: A Review. Food Bioprocess Technol..

[21] Sajid M., Khaled Nazal M., Rutkowska M., Szczepańska N., Namieśnik J., Płotka-Wasylka J. (2019). Solid Phase Microextraction: Apparatus, Sorbent Materials, and Application. Crit. Rev. Anal. Chem..

[22] Vas G., Vékey K. (2004). Solid-Phase Microextraction: A Powerful Sample Preparation Tool Prior to Mass Spectrometric Analysis. J. Mass Spectrom..

[23] Cong-Cong X.U., Wang B., Yi-Qiong P.U., Jian-Sheng T., Tong Z. (2017). Advances in Extraction and Analysis of Phenolic Compounds from Plant Materials. Chin. J. Nat. Med..

[24] Nagashima F., Asakawa Y. (2011). Terpenoids and Bibenzyls from Three Argentine Liverworts. Molecules.

[25] Linde J., Combrinck S., van Vuuren S., van Rooy J., Ludwiczuk A., Mokgalaka N. (2016). Volatile Constituents and Antimicrobial Activities of Nine South African Liverwort Species. Phytochem. Lett..

[26] Veljić M., Ćirić A., Soković M., Janaćković P., Marin P.D. (2010). Antibacterial and Antifungal Activity of the Liverwort (*Ptilidium pulcherrimum*) Methanol Extract. Arch. Biol. Sci..

[27] Asakawa Y., Ludwiczuk A., Nagashima F. (2013). Phytochemical and Biological Studies of Bryophytes. Phytochemistry.

[28] Tan M.C., Tan C.P., Ho C.W. (2013). Effects of Extraction Solvent System, Time and Temperature on Total Phenolic Content of Henna (Lawsonia Inermis) Stems. Int. Food Res. J..

[29] Ritchie R.J. (2006). Consistent Sets of Spectrophotometric Chlorophyll Equations for Acetone, Methanol and Ethanol Solvents. Photosynth. Res..

[30] Wesołowska A., Grzeszczuk M., Jadczak D. (2014). Comparison of Chemical Compositions of Essential Oils Isolated by Hydrodistillation from Wild Thyme (*Thymus Serpyllum* L.) with Use of Deryng and Clevenger Apparatus. Herba Pol..

[31] Wawrzyniak R., Wasiak W., Jasiewicz B., Bączkiewicz A., Buczkowska K. (2021). Chemical Fingerprinting of Cryptic Species and Genetic Lineages of *Aneura Pinguis* (L.) Dumort. (Marchantiophyta, Metzgeriidae). Molecules.

[32] Wawrzyniak R., Wasiak W., Bączkiewicz A., Buczkowska K. (2014). Volatile Compounds in Cryptic Species of the *Aneura pinguis* Complex and *Aneura Maxima* (Marchantiophyta, Metzgeriidae). Phytochemistry.

