# Supplementary material for: Comparison of Extraction Techniques for the Determination of Volatile Organic Compounds in Liverwort Samples
Source: Molecules. 2022 May 3;27(9):2911. doi: 10.3390/molecules27092911 (PMC9099673; doi:10.3390/molecules27092911)
Supplement: Supplementary file 1 [file molecules-27-02911-s001.zip › molecules-1708178-supplementary.pdf]

molecules

Supplementary Material

Comparison of extraction techniques for the determination of volatile organic compounds in liverwort samples

Małgorzata Guzowska [1]*, Wiesław Wasiak [1] and Rafał Wawrzyniak [1]

Table S1. Volatile compounds detected in the samples analysed by SPME and HD

No.	Compounds	RI[a]	RI[b] (Lit.)	Code[c]		
				SPME	HD1	HD2
1	pentanal	<700	701	0.11 ± 0.02	-	-
2	3-methyl-1-butanol	768	730	0.01 (*)	-	-
3	hexanal	834	782	0.01 (*)	-	-
4	107[M+](8) 91(100) 106(58)	902	-	0.57 ± 0.03	-	-
5	α-pinene	952	939	0.03 ± 0.02	-	-
6	β-pinene	1004	980	0.62 ± 0.02	-	-
7	limonene	1048	1031	0.51 ± 0.01	-	-
8	benzeneacetaldehyde	1093	1044	0.19 ± 0.02	-	-
9	1-octen-3-yl-acetate	1123	1111	0.01 ± (*)	-	0.05 ± 0.01
10	benzeneethanol	1156	1117	0.01 ± (*)	0.12 ± 0.02	0.25 ± 0.04
11	160[M+](49) 145(100) 117(34)	1224	-	3.89 ± 0.03	0.54 ± 0.03	0.89 ± 0.03
12	β-cyclocitral	1257	1220	1.35 ± 0.03	0.09 ± 0.02	0.13 ± 0.02
13	1,2-dihydro-6-methylnaphthalene	1295	1238	0.02 ± 0.01	0.01 ± (*)	0.02 ± 0.01
14	207[M+](18) 121(100) 93(71)	1332	-	0.08 ± 0.02	-	-
15	δ-elemene	1344	1339	0.47 ± 0.03	0.03 ± 0.01	0.04 ± 0.01
16	methylnaphthalene	1359	1359	0.07 ± 0.01	0.02 ± 0.01	0.03 ± 0.01
17	202[M+](18) 81(100) 96(83)	1364	-	0.07 ± 0.02	0.16 ± 0.03	0.24 ± 0.04
18	anastreptene	1379	1379	6.92 ± 0.02	14.67 ± 0.06	15.74 ± 0.08
19	204[M+](25) 105(100) 161(83)	1382	-	0.48 ± 0.01	0.04 ± 0.01	0.06 ± 0.01
20	202[M+](5) 159(100) 91(95)	1387	-	0.16 ± 0.02	0.01 ± (*)	-
21	202[M+](5) 143(100) 128(92)	1392	-	1.56 ± 0.02	0.02 ± 0.01	0.06 ± 0.02
22	β-elemene	1405	1387	0.38 ± 0.02	0.06 ± 0.01	0.08 ± 0.02
23	202[M+](2) 143(100) 128(93)	1418	-	0.20 ± 0.02	-	-
24	α-gurjunene	1423	1430	0.19 ± 0.02	0.10 ± 0.02	0.10 ± 0.03
25	202[M+](2) 145(100) 160(35)	1426	-	0.35 ± 0.02	-	-
26	β-gurjunene	1431	1473	0.15 ± 0.02	0.11 ± 0.03	0.11 ± 0.03
27	204[M+](12) 159(100) 105(95)	1436	-	0.14 ± 0.02	-	-
28	(-)-aristolene	1439	1428	0.27 ± 0.02	0.06 ± 0.01	0.06 ± 0.01
29	γ-gurjunene	1444	1430	0.02 ± 0.01	0.12 ± 0.03	0.15 ± 0.02
30	γ-maaliene	1449	1433	0.86 ± 0.01	0.08 ± 0.02	0.02 ± 0.01
31	α-maaliene	1455	1442	0.39 ± 0.02	0.21 ± 0.04	0.18 ± 0.03
32	alloaromadendrene	1459	1452	4.12 ± 0.02	0.07 ± 0.01	0.05 ± 0.01
33	selina-5,11-diene	1470	1454	0.91 ± 0.02	0.13 ± 0.02	0.18 ± 0.03
34	202(20)[M]+ 105(100) 159(68)	1475	-	0.15 ± 0.01	0.39 ± 0.02	0.59 ± 0.05
35	202(30)[M]+ 159(100) 131(75)	1479	-	0.61 ± 0.02	0.06 ± 0.01	0.05 ± 0.01
36	dehydroaromadendrene	1482	1463	0.65 ± 0.01	0.83 ± 0.04	1.22 ± 0.06
37	204[M+](1) 142(100) 141(78)	1495	-	0.16 ± 0.02	0.61 ± 0.02	0.57 ± 0.04
38	β-guaiene	1505	1473	0.02 ± 0.01	0.16 ± 0.02	0.27 ± 0.04
39	germacrene D	1510	1480	3.47 ± 0.03	0.44 ± 0.05	0.59 ± 0.04
40	ledene	1516	1487	-	0.04 ± 0.01	0.13 ± 0.03

41	bicyclogermacrene	1521	1500	4.39 ± 0.08	14.13 ± 0.10	14.77 ± 0.07
42	220[M+](5) 148(100) 133(95)	1525	-	0.18 ± 0.01	0.04 ± 0.01	0.04 ± 0.01
43	cuparene	1538	1505	0.72 ± 0.01	1.12 ± 0.03	1.41 ± 0.04
44	204[M+](22) 93(100) 105(68)	1543	-	0.09 ± 0.02	-	-
45	202[M+](12) 157(100) 142(62)	1554	-	0.34 ± 0.02	0.31 ± 0.02	0.19 ± 0.03
46	204[M+](18) 155(100) 119(82)	1558	-	0.01 ± (*)	0.04 ± 0.01	0.03 ± 0.01
47	200[M]+ (72) 143(100) 129(93)	1571	-	0.25 ± 0.02	0.28 ± 0.03	0.37 ± 0.03
48	200[M+](73) 143(100) 157(97)	1579	-	0.04 ± 0.01	0.08 ± 0.02	0.1 ± 0.02
49	nerolidol	1586	1583	0.34 ± 0.02	0.06 ± 0.01	0.06 ± 0.01
50	cadina-3,9-diene	1591	-	0.16 ± 0.02	0.16 ± 0.02	0.01 ± (*)
51	4,5,9,10-dehydro-isolongifolene	1592	-	-	1.2 ± 0.04	0.27 ± 0.03
52	maaliol	1609	-	-	1.09 ± 0.03	0.13 ± 0.03
53	1,4-dimethyl-azulene	1616	-	41.89 ± 0.03	15.74 ± 0.04	18.18 ± 0.06
54	(+)-spathulenol	1619	-	-	0.90 ± 0.01	0.84 ± 0.04
55	(-)-globulol	1631		0.24 ± 0.02	0.64 ± 0.03	0.61 ± 0.02
56	220[M+](5) 132(100) 43(96)	1637	-	0.73 ± 0.02	0.86 ± 0.02	0.72 ± 0.04
57	202[M+](2) 129(100) 172(72)	1655	-	1.83 ± 0.02	5.01 ± 0.04	3.39 ± 0.05
58	isospathulenol	1667	1631	0.33 ± 0.02	0.48 ± 0.03	0.48 ± 0.02
59	218[M+](2) 71(100) 57(93)	1674	-	0.39 ±0.02	-	-
60	220[M+](2) 161(100) 121(39)	1678	-	-	0.13 ± 0.03	0.09 ± 0.02
61	218[M+](2) 77(100) 182(33)	1684	-	-	0.09 ± 0.02	0.07 ± 0.01
62	220[M+](5) 129(100) 144(92)	1689	-	0.01 ± (*)	-	0.02 ± 0.01
63	220[M+](31) 159(100) 105(81)	1695	-	0.27 ± 0.01	0.12 ± 0.03	0.07 ± 0.01
64	202[M+](92) 143(100) 128(78)	1710	-	0.03 ± 0.01	-	0.04 ± 0.01
65	220[M+](11) 109(100) 121(51)	1722	-	0.05 ± 0.01	0.92 ± 0.03	0.89 ± 0.03
66	220[M+](13) 159(100) 145(89)	1744	--	0.12 ± 0.02	0.45 ± 0.05	0.34 ± 0.05
67	220[M+](6) 161(100) 105(73)		-	-	0.15 ± 0.02	0.05 ± 0.01
68	4-(cyclopent-1-enyl)benzoic acid methyl ester	1784	-	14.89 ± 0.03	22.32 ± 0.04	23.69 ± 0.04
69	216[M+](10) 202(100) 159(96)	1791	-	0.12 ± 0.02	-	-
70	216[M+](3) 202(100) 143(81)	1795	-	0.02 ± 0.01	-	-
71	germacra-4(15),5,10(14)-trien-1-alpha-ol	1801	-	0.41 ± 0.03	1.49 ± 0.03	1.19 ± 0.03
72	1,4-dimethyl-7-(1-methylethyl)-azulene	1823	-	0.03 ± 0.02	-	-
73	methyl 2-methylazulene-1-carboxylate	2025	-	0.11 ± 0.02	7.59 ± 0.04	4.42 ± 0.04
	Total			98.17 ± 1.09	94.58 ± 1.31	94.83 ± 1.51
	% Identified			85.84 ± 0.69	84.27 ± 0.88	85.96 ± 0.97
	Including:					
	Aliphatics			0.14 ± 0.02	-	0.05 ± 0.01
	Aromatics			15.75 ± 0.10	22.47 ± 0.70	24.99 ± 0.10
	Monoterpene hydrocarbons			1.16 ± 0.05	-	-
	Monoterpenoide hydrocarbons			1.35 ± 0.03	0.09 ± 0.20	0.13 ± 0.02
	Sesquiterpene hydrocarbons			66.42 ± 0.42	50.95 ± 0.64	55.25 ± 0.68
	Sesquiterpenoide hydrocarbons			1.02 ± 0.08	10.76 ± 0.15	6.54 ± 0.16

* less than 0.01%. [a] Retention index on Quadex 007-5MS column.[b] NIST 11 library; [c] For abbreviations of samples see Table 1. ± standard deviation

Table S2. Volatile compounds detected in the samples extracted by n-hexane

No.	Compounds	RI[a]	Code[b]		
			SLE1-1	SLE1-2	SLE1-3
1	pentanal	<700	-	-	-
2	3-methyl-1-butanol	768	-	-	-
3	hexanal	834	-	-	-
4	107[M+](8) 91(100) 106(58)	902	-	-	-
5	α-pinene	952	-	-	-
6	β-pinene	1004	-	-	-
7	limonene	1048	-	-	-
8	benzeneacetaldehyde	1093	-	-	-
9	1-octen-3-yl-acetate	1123	-	-	-
10	benzeneethanol	1156	-	-	-
11	160[M+](49) 145(100) 117(34)	1224	0.87 ± 0.03	0.86 ± 0.04	0.95 ± 0.04
12	β-cyclocitral	1257	0.12 ± 0.02	0.08 ± 0.02	0.05 ± 0.01
13	1,2-dihydro-6-methylnaphthalene	1295	-	-	-
14	207[M+](18) 121(100) 93(71)	1332	-	0.07 ± 0.01	0.08 ± 0.01
15	δ-elemene	1344	-	-	0.08 ± 0.01
16	methylnaphthalene	1359	-	-	0.04 ± 0.01
17	202[M+](18) 81(100) 96(83)	1364	-	-	0.08 ± 0.01
18	anastreptene	1379	6.94 ± 0.03	4.68 ± 0.04	4.68 ± 0.05
19	204[M+](25) 105(100) 161(83)	1382	-	-	-
20	202[M+](5) 159(100) 91(95)	1387	1.62 ± 0.02	1.39 ± 0.03	0.95 ± 0.04
21	202[M+](5) 143(100) 128(92)	1392	-	-	-
22	β-elemene	1405	0.17 ± 0.02	0.17 ± 0.02	0.17 ± 0.02
23	202[M+](2) 143(100) 128(93)	1418	-	-	-
24	α-gurjunene	1423	-	-	-
25	202[M+](2) 145(100) 160(35)	1426	-	-	-
26	β-gurjunene	1431	-	-	-
27	204[M+](12) 159(100) 105(95)	1436	-	-	-
28	(-)-aristolene	1439	-	-	-
29	γ-gurjunene	1444	-	-	-
30	γ-maaliene	1449	-	-	-
31	α-maaliene	1455	-	-	-
32	alloaromadendrene	1459	-	-	-
33	selina-5,11-diene	1470	-	-	-
34	202(20)[M]+ 105(100) 159(68)	1475	-	0.08 ± 0.01	0.10 ± 0.03
35	202(30)[M]+ 159(100) 131(75)	1479	-	-	-
36	dehydroaromadendrene	1482	0.19 ± 0.03	0.29 ± 0.04	0.29 ± 0.04
37	204[M+](1) 142(100) 141(78)	1495	-	-	-
38	β-guaiene	1505	-	-	-
39	germacrene D	1510	-	-	-
40	ledene	1516	-	-	-
41	bicyclogermacrene	1521	7.78 ± 0.04	12.01 ± 0.04	13.41 ± 0.04
42	220[M+](5) 148(100) 133(95)	1525		-	-
43	cuparene	1538	0.16 ± 0.03	0.26 ± 0.02	0.29 ± 0.04
44	204[M+](22) 93(100) 105(68)	1543	-	-	-
45	202[M+](12) 157(100) 142(62)	1554	-	-	-
46	204[M+](18) 155(100) 119(82)	1558	-	-	-
47	200[M]+ (72) 143(100) 129(93)	1571	-	-	-
48	200[M+](73) 143(100) 157(97)	1579	-	-	-
49	nerolidol	1586	0.45 ± 0.03	0.38 ± 0.03	0.31 ± 0.04
50	cadina-3,9-diene	1591	-	-	-
51	4,5,9,10-dehydro-isolongifolene	1592	0.27 ± 0.02	0.36 ± 0.03	0.38 ± 0.04
52	maaliol	1609	-	-	-
53	1,4-dimethyl-azulene	1616	59.33 ± 0.14	53.53 ± 0.11	48.88 ± 0.09
54	(+)-spathulenol	1619	-	-	-

55	(-)-globulol	1631	-	-	-
56	220[M+](5) 132(100) 43(96)	1637	-	-	-
57	202[M+](2) 129(100) 172(72)	1655	-	0.35 ± 0.02	0.71 ± 0.04
58	isospathulenol	1667	-	0.04 ± 0.01	0.04 ± 0.01
59	218[M+](2) 71(100) 57(93)	1674	-	-	-
60	220[M+](2) 161(100) 121(39)	1678	-	-	-
61	218[M+](2) 77(100) 182(33)	1684	-	-	-
62	220[M+](5) 129(100) 144(92)	1689	-	-	-
63	220[M+](31) 159(100) 105(81)	1695	-	-	-
64	202[M+](92) 143(100) 128(78)	1710	-	-	-
65	220[M+](11) 109(100) 121(51)	1722	-	-	-
66	220[M+](13) 159(100) 145(89)	1744	-	-	-
67	220[M+](6) 161(100) 105(73)		-	-	-
68	4-(cyclopent-1-enyl)benzoic acid methyl ester	1784	21.61 ± 0.06	23.20 ± 0.04	23.30 ± 0.06
69	216[M+](10) 202(100) 159(96)	1791	-	-	-
70	216[M+](3) 202(100) 143(81)	1795	-	-	-
71	germacra-4(15),5,10(14)-trien-1-alpha-ol	1801	-	-	-
72	1,4-dimethyl-7-(1-methylethyl)-azulene	1823	-	-	-
73	methyl 2-methylazulene-1-carboxylate	2025	-	0.18 ± 0.04	0.20 ± 0.03
	Total		99.51 ± 0.47	97.93 ± 0.55	95.00 ± 0.66
	% Identified		97.02 ± 0.42	95.30 ± 0.46	92.13 ± 0.49
	Including:				
	Aliphatics		-	-	-
	Aromatics		21.61 ± 0.06	23.20 ± 0.04	23.34 ± 0.07
	Monoterpene hydrocarbons		-	-	
	Monoterpenoide hydrocarbons		0.12 ± 0.02	0.08 ± 0.02	0.05 ± 0.01
	Sesquiterpene hydrocarbons		74.84 ± 0.31	71.30 ± 0.30	68.19 ± 0.33
	Sesquiterpenoide hydrocarbons		0.45 ± 0.03	0.60 ± 0.08	0.55 ± 0.08

[a] Retention index on Quadex 007-5MS column. [b] For abbreviations of samples see Table 1. ± standard deviation

Table S3. Volatile compounds detected in the samples extracted by diethyl ether

No.	Compounds	RI[a]	Code[b]		
			SLE2-1	SLE2-2	SLE2-3
1	pentanal	<700	-	-	-
2	3-methyl-1-butanol	768	-	-	-
3	hexanal	834	-	-	-
4	107[M+](8) 91(100) 106(58)	902	-	-	-
5	α-pinene	952	-	-	-
6	β-pinene	1004	-	-	-
7	limonene	1048	-	-	-
8	benzeneacetaldehyde	1093	-	-	-
9	1-octen-3-yl-acetate	1123	-	-	-
10	benzeneethanol	1156	-	-	-
11	160[M+](49) 145(100) 117(34)	1224	0.81 ± 0.04	1.14 ± 0.05	1.14 ± 0.03
12	β-cyclocitral	1257	-	-	-
13	1,2-dihydro-6-methylnaphthalene	1295	-	-	-
14	207[M+](18) 121(100) 93(71)	1332	-	-	-
15	δ-elemene	1344	-	-	-
16	methylnaphthalene	1359	-	-	-
17	202[M+](18) 81(100) 96(83)	1364	-	-	-
18	anastreptene	1379	6.82 ± 0.06	8.61 ± 0.08	14.65 ± 0.05
19	204[M+](25) 105(100) 161(83)	1382	-	-	-
20	202[M+](5) 159(100) 91(95)	1387	-	-	-
21	202[M+](5) 143(100) 128(92)	1392	0.70 ± 0.03	0.59 ± 0.03	0.31 ± 0.03
22	β-elemene	1405	-	-	-
23	202[M+](2) 143(100) 128(93)	1418	-	-	-
24	α-gurjunene	1423	-	-	-
25	202[M+](2) 145(100) 160(35)	1426	-	-	-
26	β-gurjunene	1431	-	-	-
27	204[M+](12) 159(100) 105(95)	1436	-	-	-
28	(-)-aristolene	1439	-	-	-
29	γ-gurjunene	1444	-	-	-
30	γ-maaliene	1449	-	-	-
31	α-maaliene	1455	-	-	-
32	alloaromadendrene	1459	-	-	-
33	selina-5,11-diene	1470	-	-	-
34	202(20)[M]+ 105(100) 159(68)	1475	-	-	-
35	202(30)[M]+ 159(100) 131(75)	1479	-	-	-
36	dehydroaromadendrene	1482	-	-	-
37	204[M+](1) 142(100) 141(78)	1495	-	-	-
38	β-guaiene	1505	-	-	-
39	germacrene D	1510	-	-	-
40	ledene	1516	-	-	-
41	bicyclogermacrene	1521	7.38 ± 0.06	10.94 ± 0.04	17.59 ± 0.05
42	220[M+](5) 148(100) 133(95)	1525	-	-	-
43	cuparene	1538	-	-	-
44	204[M+](22) 93(100) 105(68)	1543	-	-	-
45	202[M+](12) 157(100) 142(62)	1554	-	-	-
46	204[M+](18) 155(100) 119(82)	1558	-	-	-
47	200[M]+ (72) 143(100) 129(93)	1571	-	-	-
48	200[M+](73) 143(100) 157(97)	1579	-	-	-
49	nerolidol	1586	-	-	-
50	cadina-3,9-diene	1591	-	-	-
51	4,5,9,10-dehydro-isolongifolene	1592	0.34 ± 0.04	0.22 ± 0.02	0.15 ± 0.02
52	maaliol	1609	-	-	-
53	1,4-dimethyl-azulene	1616	47.19 ± 0.08	45.89 ± 0.08	43.52 ± 0.08
54	(+)-spathulenol	1619	-	-	-

55	(-)-globulol	1631	-	-	-
56	220[M+](5) 132(100) 43(96)	1637	-	-	-
57	202[M+](2) 129(100) 172(72)	1655	-	-	-
58	isospathulenol	1667	-	-	-
59	218[M+](2) 71(100) 57(93)	1674	-	-	-
60	220[M+](2) 161(100) 121(39)	1678	-	-	-
61	218[M+](2) 77(100) 182(33)	1684	-	-	-
62	220[M+](5) 129(100) 144(92)	1689	-	-	-
63	220[M+](31) 159(100) 105(81)	1695	-	-	-
64	202[M+](92) 143(100) 128(78)	1710	-	-	-
65	220[M+](11) 109(100) 121(51)	1722	-	-	-
66	220[M+](13) 159(100) 145(89)	1744	-	-	-
67	220[M+](6) 161(100) 105(73)		-	-	-
68	4-(cyclopent-1-enyl)benzoic acid methyl ester	1784	27.22 ± 0.04	26.09 ± 0.04	19.45 ± 0.05
69	216[M+](10) 202(100) 159(96)	1791	-	-	-
70	216[M+](3) 202(100) 143(81)	1795	-	-	-
71	germacra-4(15),5,10(14)-trien-1-alpha-ol	1801	-	-	-
72	1,4-dimethyl-7-(1-methylethyl)-azulene	1823	-	-	-
73	methyl 2-methylazulene-1-carboxylate	2025	1.02 ± 0.02	1.48 ± 0.05	1.48 ± 0.02
	Total		91.48 ± 0.37	94.96 ± 0.39	98.29 ± 0.33
	% Identified		89.97 ± 0.30	93.23 ± 0.31	96.84 ± 0.27
	Including:				
	Aliphatics		-	-	-
	Aromatics		27.22 ± 0.04	26.09 ± 0.04	19.45 ± 0.05
	Monoterpene hydrocarbons		-	-	-
	Monoterpenoide hydrocarbons		-	-	-
	Sesquiterpene hydrocarbons		61.73 ± 0.24	65.66 ± 0.22	75.91 ± 0.20
	Sesquiterpenoide hydrocarbons		1.02 ± 0.02	1.48 ± 0.05	1.48 ± 0.02

[a] Retention index on Quadex 007-5MS column. [b] For abbreviations of samples see Table 1. ± standard deviation

Table S4. Volatile compounds detected in the samples extracted by methylene chloride

No.	Compounds	RI[a]	Code[b]		
			SLE3-1	SLE3-2	SLE3-3
1	pentanal	<700	-	-	-
2	3-methyl-1-butanol	768	-	-	-
3	hexanal	834	-	-	-
4	107[M+](8) 91(100) 106(58)	902	-	-	-
5	α-pinene	952	-	-	-
6	β-pinene	1004	-	-	-
7	limonene	1048	-	-	-
8	benzeneacetaldehyde	1093	-	-	-
9	1-octen-3-yl-acetate	1123	-	-	-
10	benzeneethanol	1156	-	-	-
11	160[M+](49) 145(100) 117(34)	1224	0.88 ± 0.03	0.95 ± 0.03	0.96 ± 0.03
12	β-cyclocitral	1257	-	-	-
13	1,2-dihydro-6-methylnaphthalene	1295	-	-	-
14	207[M+](18) 121(100) 93(71)	1332	-	-	-
15	δ-elemene	1344	-	-	-
16	methylnaphthalene	1359	-	-	-
17	202[M+](18) 81(100) 96(83)	1364	-	-	-
18	anastreptene	1379	8.82 ± 0.04	10.01 ± 0.04	10.74 ± 0.04
19	204[M+](25) 105(100) 161(83)	1382	-	-	-
20	202[M+](5) 159(100) 91(95)	1387	-	-	-
21	202[M+](5) 143(100) 128(92)	1392	0.82 ± 0.03	0.85 ± 0.03	0.85 ± 0.02
22	β-elemene	1405	-	-	-
23	202[M+](2) 143(100) 128(93)	1418	-	-	-
24	α-gurjunene	1423	-	-	-
25	202[M+](2) 145(100) 160(35)	1426	-	-	-
26	β-gurjunene	1431	-	-	-
27	204[M+](12) 159(100) 105(95)	1436	-	-	-
28	(-)-aristolene	1439	-	-	-
29	γ-gurjunene	1444	-	-	-
30	γ-maaliene	1449	-	-	-
31	α-maaliene	1455	-	-	-
32	alloaromadendrene	1459	-	-	-
33	selina-5,11-diene	1470	-	-	-
34	202(20)[M]+ 105(100) 159(68)	1475	-	-	-
35	202(30)[M]+ 159(100) 131(75)	1479	-	-	-
36	dehydroaromadendrene	1482	10.23 ± 0.04	12.47 ± 0.04	12.68 ± 0.04
37	204[M+](1) 142(100) 141(78)	1495	-	-	-
38	β-guaiene	1505	-	-	-
39	germacrene D	1510	-	-	-
40	ledene	1516	-	-	-
41	bicyclogermacrene	1521	-	-	-
42	220[M+](5) 148(100) 133(95)	1525	-	-	-
43	cuparene	1538	-	-	-
44	204[M+](22) 93(100) 105(68)	1543	-	-	-
45	202[M+](12) 157(100) 142(62)	1554	-	-	-
46	204[M+](18) 155(100) 119(82)	1558	-	-	-
47	200[M]+ (72) 143(100) 129(93)	1571	-	-	-
48	200[M+](73) 143(100) 157(97)	1579	-	-	-
49	nerolidol	1586	0.56 ± 0.05	0.35 ± 0.03	0.31 ± 0.03
50	cadina-3,9-diene	1591	-	-	-
51	4,5,9,10-dehydro-isolongifolene	1592	-	-	-
52	maaliol	1609	-	-	-
53	1,4-dimethyl-azulene	1616	55.18 ± 0.08	50.69 ± 0.08	47.04 ± 0.08
54	(+)-spathulenol	1619		-	-

55	(-)-globulol	1631	-	-	-
56	220[M+](5) 132(100) 43(96)	1637	-	-	-
57	202[M+](2) 129(100) 172(72)	1655	-	-	-
58	isospathulenol	1667	-	-	-
59	218[M+](2) 71(100) 57(93)	1674	-	-	-
60	220[M+](2) 161(100) 121(39)	1678	-	-	-
61	218[M+](2) 77(100) 182(33)	1684	-	-	-
62	220[M+](5) 129(100) 144(92)	1689	-	-	-
63	220[M+](31) 159(100) 105(81)	1695	-	-	-
64	202[M+](92) 143(100) 128(78)	1710	-	-	-
65	220[M+](11) 109(100) 121(51)	1722	-	-	-
66	220[M+](13) 159(100) 145(89)	1744	-	-	-
67	220[M+](6) 161(100) 105(73)		-	-	-
68	4-(cyclopent-1-enyl)benzoic acid methyl ester	1784	18.51 ± 0.04	18.75 ± 0.04	20.79 ± 0.04
69	216[M+](10) 202(100) 159(96)	1791	-	-	-
70	216[M+](3) 202(100) 143(81)	1795	-	-	-
71	germacra-4(15),5,10(14)-trien-1-alpha-ol	1801	-	-	-
72	1,4-dimethyl-7-(1-methylethyl)-azulene	1823	-	2.44 ± 0.02	2.44 ± 0.03
73	methyl 2-methylazulene-1-carboxylate	2025	1.97 ± 0.03	2.10 ± 0.02	2.39 ± 0.02
	Total		96.97 ± 0.34	98.61 ± 0.35	98.20 ± 0.33
	% Identified		95.27 ± 0.28	96.81 ± 0.29	96.39 ± 0.28
	Including:				
	Aliphatics		-	-	-
	Aromatics		18.51 ± 0.04	18.75 ± 0.04	20.79 ± 0.04
	Monoterpene hydrocarbons		-	-	-
	Monoterpenoide hydrocarbons		-	-	-
	Sesquiterpene hydrocarbons		74.23 ± 0.16	75.61 ± 0.20	72.90 ± 0.19
	Sesquiterpenoide hydrocarbons		2.53 ± 0.08	2.45 ± 0.05	2.70 ± 0.05

[a] Retention index on Quadex 007-5MS column. [b] For abbreviations of samples see Table 1. ± standard deviation

Table S5. Volatile compounds detected in the samples extracted by ethyl acetate

No.	Compounds	RI[a]	Code[b]		
			SLE4-1	SLE4-2	SLE4-3
1	pentanal	<700	-	-	-
2	3-methyl-1-butanol	768	-	-	-
3	hexanal	834	-	-	-
4	107[M+](8) 91(100) 106(58)	902	-	-	-
5	α-pinene	952	-	-	-
6	β-pinene	1004	-	-	-
7	limonene	1048	-	-	-
8	benzeneacetaldehyde	1093	-	-	-
9	1-octen-3-yl-acetate	1123	-	-	-
10	benzeneethanol	1156	0.09 ± 0.02	0.11 ± 0.02	0.18 ± 0.03
11	160[M+](49) 145(100) 117(34)	1224	1.12 ± 0.03	1.14 ± 0.03	1.18 ± 0.02
12	β-cyclocitral	1257	0.09 ± 0.01	0.11 ± 0.02	0.17 ± 0.02
13	1,2-dihydro-6-methylnaphthalene	1295	-	-	-
14	207[M+](18) 121(100) 93(71)	1332	-	-	-
15	δ-elemene	1344	-	-	-
16	methylnaphthalene	1359	-	-	-
17	202[M+](18) 81(100) 96(83)	1364	-	-	-
18	anastreptene	1379	7.00 ± 0.03	7.84 ± 0.03	7.85 ± 0.03
19	204[M+](25) 105(100) 161(83)	1382	-	-	-
20	202[M+](5) 159(100) 91(95)	1387	-	-	-
21	202[M+](5) 143(100) 128(92)	1392	0.14 ± 0.02	0.16 ± 0.02	0.16 ± 0.02
22	β-elemene	1405	-	-	-
23	202[M+](2) 143(100) 128(93)	1418	-	-	-
24	α-gurjunene	1423	-	-	-
25	202[M+](2) 145(100) 160(35)	1426	-	-	-
26	β-gurjunene	1431	-	-	-
27	204[M+](12) 159(100) 105(95)	1436	-	-	-
28	(-)-aristolene	1439	-	-	-
29	γ-gurjunene	1444	-	-	-
30	γ-maaliene	1449	-	-	-
31	α-maaliene	1455	-	-	-
32	alloaromadendrene	1459	-	-	-
33	selina-5,11-diene	1470	-	-	-
34	202(20)[M]+ 105(100) 159(68)	1475	-	-	-
35	202(30)[M]+ 159(100) 131(75)	1479	-	-	0.29 ± 0.03
36	dehydroaromadendrene	1482	-	-	-
37	204[M+](1) 142(100) 141(78)	1495	-	-	-
38	β-guaiene	1505	-	-	-
39	germacrene D	1510	-	-	-
40	ledene	1516	-	-	-
41	bicyclogermacrene	1521	7.32 ± 0.03	10.88 ± 0.03	12.24 ± 0.02
42	220[M+](5) 148(100) 133(95)	1525	-	-	-
43	cuparene	1538	-	-	-
44	204[M+](22) 93(100) 105(68)	1543	-	-	-
45	202[M+](12) 157(100) 142(62)	1554	-	-	-
46	204[M+](18) 155(100) 119(82)	1558	-	-	-
47	200[M]+ (72) 143(100) 129(93)	1571	-	-	-
48	200[M+](73) 143(100) 157(97)	1579	-	-	-
49	nerolidol	1586	0.56 ± 0.05	0.12 ± 0.01	0.12 ± 0.01
50	cadina-3,9-diene	1591	-	-	-
51	4,5,9,10-dehydro-isolongifolene	1592	0.56 ± 0.02	0.70 ± 0.03	0.70 ± 0.02
52	maaliol	1609		-	-
53	1,4-dimethyl-azulene	1616	39.94 ± 0.06	36.01 ± 0.06	35.99 ± 0.06
54	(+)-spathulenol	1619		-	-

55	(-)-globulol	1631	-	-	-
56	220[M+](5) 132(100) 43(96)	1637	0.47 ± 0.03	0.40 ± 0.03	0.39 ± 0.03
57	202[M+](2) 129(100) 172(72)	1655	1.23 ± 0.02	1.14 ± 0.02	0.98 ± 0.03
58	isospathulenol	1667	-	-	-
59	218[M+](2) 71(100) 57(93)	1674	-	-	-
60	220[M+](2) 161(100) 121(39)	1678	-	-	-
61	218[M+](2) 77(100) 182(33)	1684	-	-	-
62	220[M+](5) 129(100) 144(92)	1689	-	-	-
63	220[M+](31) 159(100) 105(81)	1695	-	-	-
64	202[M+](92) 143(100) 128(78)	1710	-	-	-
65	220[M+](11) 109(100) 121(51)	1722	-	0.40 ± 0.04	0.73 ± 0.02
66	220[M+](13) 159(100) 145(89)	1744	-	-	-
67	220[M+](6) 161(100) 105(73)		-	-	-
68	4-(cyclopent-1-enyl)benzoic acid methyl ester	1784	37.68 ± 0.04	35.66 ± 0.04	30.82 ± 0.04
69	216[M+](10) 202(100) 159(96)	1791	-	-	-
70	216[M+](3) 202(100) 143(81)	1795	-	-	-
71	germacra-4(15),5,10(14)-trien-1-alpha-ol	1801	0.56 ± 0.03	0.73 ± 0.03	0.85 ± 0.03
72	1,4-dimethyl-7-(1-methylethyl)-azulene	1823	-	-	-
73	methyl 2-methylazulene-1-carboxylate	2025	1.61 ± 0.02	3.45 ± 0.04	3.45 ± 0.03
	Total		97.81 ± 0.36	99.14 ± 0.43	96.10 ± 0.48
	% Identified		94.85 ± 0.26	95.61 ± 0.31	92.37 ± 0.31
	Including:				
	Aliphatics		-	-	-
	Aromatics		37.77 ± 0.06	35.77 ± 0.06	31.00 ± 0.07
	Monoterpene hydrocarbons		-	-	
	Monoterpenoide hydrocarbons		0.09 ± 0.01	0.11 ± 0.02	0.17 ± 0.02
	Sesquiterpene hydrocarbons		55.38 ± 0.17	56.16 ± 0.18	57.63 ± 0.16
	Sesquiterpenoide hydrocarbons		1.61 ± 0.02	3.57 ± 0.05	3.57 ± 0.06

[a] Retention index on Quadex 007-5MS column. [b] For abbreviations of samples see Table 1. ± standard deviation

Table S6. Volatile compounds detected in the samples extracted by methanol

No.	Compounds	RI[a]	Code[b]		
			SLE5-1	SLE5-2	SLE5-3
1	pentanal	<700	-	-	-
2	3-methyl-1-butanol	768	-	-	-
3	hexanal	834	-	-	-
4	107[M+](8) 91(100) 106(58)	902	-	-	-
5	α-pinene	952	-	-	-
6	β-pinene	1004	-	-	-
7	limonene	1048	-	-	-
8	benzeneacetaldehyde	1093	-	-	-
9	1-octen-3-yl-acetate	1123	-	-	-
10	benzeneethanol	1156	-	-	-
11	160[M+](49) 145(100) 117(34)	1224	0.49 ± 0.03	0.78 ± 0.02	0.78 ± 0.02
12	β-cyclocitral	1257	-	-	-
13	1,2-dihydro-6-methylnaphthalene	1295	-	-	-
14	207[M+](18) 121(100) 93(71)	1332	-	-	-
15	δ-elemene	1344	-	-	-
16	methylnaphthalene	1359	-	-	-
17	202[M+](18) 81(100) 96(83)	1364	-	-	-
18	anastreptene	1379	11.37 ± 0.03	13.16 ± 0.03	13.16 ± 0.03
19	204[M+](25) 105(100) 161(83)	1382	-	-	-
20	202[M+](5) 159(100) 91(95)	1387	-	-	-
21	202[M+](5) 143(100) 128(92)	1392	0.99 ± 0.02	1.11 ± 0.03	1.11 ± 0.02
22	β-elemene	1405	-	-	-
23	202[M+](2) 143(100) 128(93)	1418	-	-	-
24	α-gurjunene	1423	-	-	-
25	202[M+](2) 145(100) 160(35)	1426	-	-	-
26	β-gurjunene	1431	-	-	-
27	204[M+](12) 159(100) 105(95)	1436	-	-	-
28	(-)-aristolene	1439	-	-	-
29	γ-gurjunene	1444	-	-	-
30	γ-maaliene	1449	-	-	-
31	α-maaliene	1455	-	-	-
32	alloaromadendrene	1459	-	-	-
33	selina-5,11-diene	1470	-	-	-
34	202(20)[M]+ 105(100) 159(68)	1475	-	-	-
35	202(30)[M]+ 159(100) 131(75)	1479	0.28 ± 0.02	0.33 ± 0.02	0.33 ± 0.02
36	dehydroaromadendrene	1482	0.30 ± 0.03	0.38 ± 0.02	0.53 ± 0.02
37	204[M+](1) 142(100) 141(78)	1495	-	-	-
38	β-guaiene	1505	-	0.48 ± 0.02	0,48 ± 0.02
39	germacrene D	1510	-	-	-
40	ledene	1516	-	-	-
41	bicyclogermacrene	1521	16.05 ± 0.03	16.87 ± 0.03	16.87 ± 0.03
42	220[M+](5) 148(100) 133(95)	1525	-	-	-
43	cuparene	1538	-	-	-
44	204[M+](22) 93(100) 105(68)	1543	-	-	-
45	202[M+](12) 157(100) 142(62)	1554	-	-	-
46	204[M+](18) 155(100) 119(82)	1558	-	-	-
47	200[M]+ (72) 143(100) 129(93)	1571	-	-	-
48	200[M+](73) 143(100) 157(97)	1579	-	-	-
49	nerolidol	1586	-	-	-
50	cadina-3,9-diene	1591	-	-	-
51	4,5,9,10-dehydro-isolongifolene	1592	0.17 ± 0.02	0.15 ± 0.02	0.12 ± 0.02
52	maaliol	1609	-	-	-
53	1,4-dimethyl-azulene	1616	44.75 ± 0.07	45.68 ± 0.07	45.70 ± 0.06
54	(+)-spathulenol	1619	-	-	-

55	(-)-globulol	1631	-	-	-
56	220[M+](5) 132(100) 43(96)	1637	-	-	-
57	202[M+](2) 129(100) 172(72)	1655	-	-	-
58	isospathulenol	1667	-	-	-
59	218[M+](2) 71(100) 57(93)	1674	-	-	-
60	220[M+](2) 161(100) 121(39)	1678	-	-	-
61	218[M+](2) 77(100) 182(33)	1684	-	-	-
62	220[M+](5) 129(100) 144(92)	1689	-	-	-
63	220[M+](31) 159(100) 105(81)	1695	-	-	-
64	202[M+](92) 143(100) 128(78)	1710	-	-	-
65	220[M+](11) 109(100) 121(51)	1722	-	-	-
66	220[M+](13) 159(100) 145(89)	1744	-	-	-
67	220[M+](6) 161(100) 105(73)		-	-	-
68	4-(cyclopent-1-enyl)benzoic acid methyl ester	1784	20.13 ± 0.04	16.30 ± 0.04	16.30 ± 0.03
69	216[M+](10) 202(100) 159(96)	1791	-	-	-
70	216[M+](3) 202(100) 143(81)	1795	-	-	-
71	germacra-4(15),5,10(14)-trien-1-alpha-ol	1801	-	-	-
72	1,4-dimethyl-7-(1-methylethyl)-azulene	1823	-	1.21 ± 0.02	1.21 ± 0.02
73	methyl 2-methylazulene-1-carboxylate	2025	1.77 ± 0.03	1.18 ±0.03	2.69 ± 0.02
	Total		96.30 ± 0.32	97.63 ± 0.36	99.28 ± 0.31
	% Identified		94.54 ± 0.25	95.41 ± 0.29	95.40 ± 0.25
	Including:				
	Aliphatics		-	-	-
	Aromatics		20.13 ± 0.04	16.30 ± 0.04	16.30 ± 0.03
	Monoterpene hydrocarbons		-	-	-
	Monoterpenoide hydrocarbons		-	-	-
	Sesquiterpene hydrocarbons		72.64 ± 0.18	77.93 ± 0.22	78.07 ± 0.20
	Sesquiterpenoide hydrocarbons		1.77 ± 0.03	1.18 ± 0.03	2.69 ± 0.02

[a] Retention index on Quadex 007-5MS column. [b] For abbreviations of samples see Table 1. ± standard deviation

Table S7. Volatile compounds detected in the samples analysed by MAE

No.	Compounds	RI[a]	Code[b]			
			MAE1	MAE2	MAE3	MAE4
1	pentanal	<700	-	-	-	-
2	3-methyl-1-butanol	768	-	-	-	-
3	hexanal	834	-	-	-	-
4	107[M+](8) 91(100) 106(58)	902	-	-	-	-
5	α-pinene	952	-	-	-	-
6	β-pinene	1004	-	-	-	-
7	limonene	1048	-	-	-	-
8	benzeneacetaldehyde	1093	0.20 ± 0.02	0.12 ± 0.02	0.10 ± 0.02	0.11 ± 0.03
9	1-octen-3-yl-acetate	1123	0.07 ± 0.02	0.06 ± 0.01	0.06 ± 0.01	0.04 ± 0.01
10	benzeneethanol	1156	-	-	0.08 ± 0.02	-
11	160[M+](49) 145(100) 117(34)	1224	0.13 ± 0.03	0.65 ± 0.03	0.09 ± 0.02	0.22 ± 0.03
12	β-cyclocitral	1257	0.11 ± 0.02	0.08 ± 0.01	0.07 ± 0.02	-
13	1,2-dihydro-6-methylnaphthalene	1295	-	-	-	-
14	207[M+](18) 121(100) 93(71)	1332	-	-	-	-
15	δ-elemene	1344	-	-	-	-
16	methylnaphthalene	1359	-	-	-	-
17	202[M+](18) 81(100) 96(83)	1364	0.04 ± 0.01	0.02 ± 0.01	-	0.02 ± 0.01
18	anastreptene	1379	7.74 ± 0.03	7.24 ± 0.04	6.12 ± 0.04	5.98 ± 0.03
19	204[M+](25) 105(100) 161(83)	1382	-	-	-	-
20	202[M+](5) 159(100) 91(95)	1387	-	-	-	-
21	202[M+](5) 143(100) 128(92)	1392	-	-	0.11 ± 0.02	-
22	β-elemene	1405	-	-	-	-
23	202[M+](2) 143(100) 128(93)	1418	-	-	-	-
24	α-gurjunene	1423	-	-	-	-
25	202[M+](2) 145(100) 160(35)	1426	-	-	-	-
26	β-gurjunene	1431	-	-	-	-
27	204[M+](12) 159(100) 105(95)	1436	-	-	-	-
28	(-)-aristolene	1439	-	-	-	-
29	γ-gurjunene	1444	-	-	-	-
30	γ-maaliene	1449	-	-	-	-
31	α-maaliene	1455	-	-	-	-
32	alloaromadendrene	1459	-	-	-	-
33	selina-5,11-diene	1470	-	-	-	-
34	202(20)[M]+ 105(100) 159(68)	1475	-	-	-	-
35	202(30)[M]+ 159(100) 131(75)	1479	-	-	-	0.11 ± 0.02
36	dehydroaromadendrene	1482	0.56 ± 0.02	11.12 ± 0.04	0.44 ± 0.03	0.11 ± 0.02
37	204[M+](1) 142(100) 141(78)	1495	0.14 ± 0.02	0.10 ± 0.02	0.12 ± 0.02	0.09 ± 0.03
38	β-guaiene	1505	-	-	-	-
39	germacrene D	1510	0.06 ± 0.01	0.03 ± 0.01	-	-
40	ledene	1516	-	-	-	-
41	bicyclogermacrene	1521	7.95 ± 0.03	-	6.48 ± 0.04	7.32 ± 0.03
42	220[M+](5) 148(100) 133(95)	1525	-	-	-	-
43	cuparene	1538	0.14 ± 0.02	0.12 ± 0.04	-	0.09 ± 0.02
44	204[M+](22) 93(100) 105(68)	1543	-	-	-	-
45	202[M+](12) 157(100) 142(62)	1554	-	-	-	-
46	204[M+](18) 155(100) 119(82)	1558	-	-	-	-
47	200[M]+ (72) 143(100) 129(93)	1571	-	-	-	-
48	200[M+](73) 143(100) 157(97)	1579	-	-	-	-
49	nerolidol	1586	-	0.32 ± 0.03	-	-
50	cadina-3,9-diene	1591	-	-	-	-
51	4,5,9,10-dehydro-isolongifolene	1592	0.15 ± 0.02	-	0.54 ± 0.03	0.11 ± 0.02
52	maaliol	1609	-	-	-	-
53	1,4-dimethyl-azulene	1616	0.25 ± 0.02	0.89 ± 0.04	0.22 ± 0.02	0.12 ± 0.02
54	(+)-spathulenol	1619	1.53 ± 0.02	-	-	-

55	(-)-globulol	1631	-	-	-	-
56	220[M+](5) 132(100) 43(96)	1637	1.00 ± 0.03	0.66 ± 0.03	0.22 ± 0.03	0.07 ± 0.01
57	202[M+](2) 129(100) 172(72)	1655	3.53 ± 0.04	5.45 ± 0.04	0.09 ± 0.02	2.22 ± 0.04
58	isospathulenol	1667	-	-	-	-
59	218[M+](2) 71(100) 57(93)	1674	-	-	-	-
60	220[M+](2) 161(100) 121(39)	1678	-	-	-	-
61	218[M+](2) 77(100) 182(33)	1684	-	-	-	-
62	220[M+](5) 129(100) 144(92)	1689	-	-	-	-
63	220[M+](31) 159(100) 105(81)	1695	-	-	-	-
64	202[M+](92) 143(100) 128(78)	1710	25.02 ± 0.04	18.17 ± 0.05	22.21 ± 0.04	21.22 ± 0.03
65	220[M+](11) 109(100) 121(51)	1722	1.44 ± 0.02	1.12 ± 0.02	0.22 ± 0.02	1.12 ± 0.02
66	220[M+](13) 159(100) 145(89)	1744	-	-	-	-
67	220[M+](6) 161(100) 105(73)		-	-	-	-
68	4-(cyclopent-1-enyl)benzoic acid methyl ester	1784	-	-	-	-
69	216[M+](10) 202(100) 159(96)	1791	-	-	-	-
70	216[M+](3) 202(100) 143(81)	1795	-	-	-	-
71	germacra-4(15),5,10(14)-trien-1-alpha-ol	1801	-	-	0.22 ± 0.03	-
72	1,4-dimethyl-7-(1-methylethyl)-azulene	1823	-	-	-	-
73	methyl 2-methylazulene-1-carboxylate	2025	20.03 ± 0.04	18.76 ± 0.04	18.23 ± 0.04	17.76 ± 0.03
	Total		70.09 ± 0.46	64.91 ± 0.48	55.62 ± 0.47	56.71± 0.40
	% Identified		38.79 ± 0.27	38.74 ± 0.28	32.56 ± 0.30	31.64 ± 0.21
	Including:					
	Aliphatics		0.07 ± 0.02	0.06 ± 0.01	0.06 ± 0.01	0.04 ± 0.01
	Aromatics		0.20 ± 0.02	0.12 ± 0.02	0.18 ± 0.04	0.11 ± 0.03
	Monoterpene hydrocarbons		-	-	-	-
	Monoterpenoide hydrocarbons		0.11 ± 0.02	0.08 ± 0.01	0.07 ± 0.02	-
	Sesquiterpene hydrocarbons		16.85 ± 0.15	19.40 ± 0.17	14.02 ± 0.19	13.73 ± 0.14
	Sesquiterpenoide hydrocarbons		21.56 ± 0.06	19.08 ± 0.07	18.23 ± 0.04	17.76 ± 0.03

[a] Retention index on Quadex 007-5MS column. [b] For abbreviations of samples see Table 1. ± standard deviation
